# Electrolyzed Oxidizing Water Modulates the Immune Response in BALB/c Mice Experimentally Infected with *Trypanosoma cruzi*

**DOI:** 10.3390/pathogens9110974

**Published:** 2020-11-23

**Authors:** Olivia Rodríguez-Morales, Juan José Cabrera-Mata, Silvia del C. Carrillo-Sánchez, Rodolfo A. Gutiérrez-Ocejo, Lidia Baylón-Pacheco, Olga L. Pérez-Reyes, José Luis Rosales-Encina, Alberto Aranda-Fraustro, Sergio Hernández-García, Minerva Arce-Fonseca

**Affiliations:** 1 Laboratory of Molecular Immunology and Proteomics, Department of Molecular Biology, National Institute of Cardiology “Ignacio Chávez”, Juan Badiano No. 1, Col. Sección XVI, Tlalpan, Mexico City 14080, Mexico; rm.olivia@gmail.com (O.R.-M.); jjcm_g@hotmail.com (J.J.C.-M.); chivy73@yahoo.com.mx (S.d.C.C.-S.); rodolfogtzocejo@gmail.com (R.A.G.-O.); 2 Department of Infectomics and Molecular Pathogenesis, Center for Research and Advanced Studies of the National Polytechnic Institute, Av. Instituto Politécnico Nacional No. 2508, Col. San Pedro Zacatenco, Gustavo A. Madero, Mexico City 07360, Mexico; baylid@hotmail.com (L.B.-P.); rosales@cinvestav.mx (J.L.R.-E.); 3 Department of Pathology, National Institute of Cardiology “Ignacio Chávez”, Juan Badiano No. 1, Col. Sección XVI, Tlalpan, Mexico City 14080, Mexico; perolgl30@gmail.com (O.L.P.-R.); arandafraustro@yahoo.com.mx (A.A.-F.); 4 Department of Cell Biology, Center for Research and Advanced Studies of the National Polytechnic Institute, Av. Instituto Politécnico Nacional No. 2508, Col. San Pedro Zacatenco, Gustavo A. Madero, Mexico City 07360, Mexico; shernandez@cinvestav.mx

**Keywords:** *Trypanosoma cruzi*, Chagas disease, murine model, electrolyzed oxidizing water, prophylaxis, therapy, immunomodulator

## Abstract

Chagas disease is a major public health problem in Latin America. The mixed Th1/Th2 immune response is required against *Trypanosoma cruzi*. Electrolyzed oxidizing water (EOW) has been shown to have germicidal efficacy. The objective of this study was to evaluate the EOW effectiveness in *T. cruzi-*infected BALB/c mice clinically, immunologically, and histologically. The severity of the infection was assessed by parasitaemia, general health condition, mortality, mega syndromes, and histological lesions. IgG, TNF-alpha, IFN-gamma, and IL-1 beta levels were quantified. The EOW administration showed a beneficial effect on parasitaemia, general physical condition, and mortality. High levels of IgG1 at 50 days postinfection were observed. Prophylactic EOW treatment was able to induce a predominantly TH1 immune response based on an IgG2a levels increase at the late acute phase, and a 10-fold increase of IFN-gamma in whole acute phase. EOW was able to control the acute phase infection as effectively as benznidazole. Splenomegaly was caused by EOW treatment and lymphadenopathy was stimulated by *T. cruzi* infection in all groups. Severe tissue damage was not prevented by EOW treatments. Moderate efficacy may be due to immunomodulatory properties and not to a direct toxic effect on the parasite.

## 1. Introduction

Chagas disease (ChD), or American trypanosomiasis, is an infection caused by the *Trypanosoma cruzi* parasite. The World Health Organization (WHO) estimates that there are approximately 6–7 million infected people worldwide, mostly in Latin America. It causes more than 10,000 deaths per year [1] and represents a loss of USD 10,000 to 11,000 per patient per year for health systems [2]. Mexico, together with Brazil and Bolivia top the list of countries with more cases reported per year [3]; however, there are no official statistics. For a long time, ChD was considered exclusive to the southern cone of America; however, the increase in migration was able to facilitate the spread to countries in Europe and North America, making it an emerging disease [4].

ChD has two phases: acute and chronic. The acute phase begins seven to ten days after infection with nonspecific clinical manifestations [5]. In less than 1% of cases, a severe form capable of causing death can develop. The chronic phase can occur in two ways: the asymptomatic (latent) and the symptomatic form. Approximately one third of these patients will develop the symptomatic form within 20 years with specific organs being affected such as heart, esophagus and/or colon [3]. 

Because *T. cruzi* develops in a circulating and intracellular form within its host, both humoral and cellular immune responses play a fundamental role in the body’s defense against this parasite. The cellular immune response is undoubtedly the most important; however, the ability of *T. cruzi* to escape and/or modulate the immune response was already described. The cellular immune response is mediated by the production of Th1-type cytokines such as Interleukin-10 (IL-10), Tumor Necrosis Factor-alpha (TNF-α) and Interferon-gamma (IFN-γ) produced by CD4+ T lymphocytes; the increase in these cytokines was associated with reduction of parasitaemia, absence or decrease of symptoms and less histological damage [6]. B lymphocytes and antibodies or immunoglobulins (Ig) are also important in the control of *T. cruzi* infection. Some IgG subclasses are responsible for the local and systemical parasite elimination by mechanisms of complement fixation, agglutination, and cytotoxicity [7,8,9]. In this regard, the normal function of various immune system cells such as B and T lymphocytes is ineffective and even beneficial to the parasite. It was also shown that *T. cruzi* is capable of triggering an autoimmune response, which seems to explain the pathophysiology of ChD [8,9,10,11].

There are currently no prophylactic measures available that are totally effective [12]. The only two drugs that were effective against ChD are nifurtimox and benznidazole, both with cure rates of 80% to 90% when used in the acute phase of the disease; however, when they are used in the chronic phase, limited and still controversial effect was shown. One of the great disadvantages is a wide range of side effects that force their interruption immediately in most cases [13]. Even more worrying, in recent years the development of drug resistance of various *T. cruzi* strains was exposed, limiting the use of these specific treatments [14]. 

In Mexico, none of these two drugs are included in the basic health scheme; therefore, they need to be imported, generating additional costs, and delaying the start of treatment up to one year [15]. Consequently, despite the proven efficacy of the current treatment, it is vital to search for new compounds or strategies that provide a better prognosis for chronic ChD, reduce costs, are easily accessible and have a less aggressive pharmacological profile. 

Electrolyzed oxidizing water (EOW) is an innovative high-level disinfectant, which has been displacing conventional products in wound cleaning and sterilization of medical instruments. EOW is produced through electrolysis of purified water plus a saturated sodium chloride solution, obtaining a solution with neutral pH and stable ion amounts. Properties of controlled concentration and chemical stability make it safe for the body’s cells, therefore it is even registered with the Federal Commission for Protection against Health Risks (COFEPRIS, for the acronym in Spanish) as a food-grade solution. In this regard, there are multiple studies and reviews describing its effectiveness against microorganisms such as *E. coli, Listeria, Salmonella*, *Bacillus anthracis*, *Pseudomonas aeruginosa*, etc. [16].

Other studies demonstrated the usefulness of EOW against intracellular organisms, such as the influenza A virus [17]. It was shown in cell cultures that both the cytopathic effect and the presence of viral antigens and genome were reduced, while preserving cellular integrity [17]. However, to date there are no studies reported in protozoa or other parasites that prove their efficacy in vivo. The mechanism of action of EOW is attributed to oxidation of the sulfhydryl groups and amino acids of the bacterial wall that affects the respiration and nutrition process, inhibition in the synthesis of proteins, breaking of the RNA chains and repression in the synthesis of cell metabolism molecules with decreased production of adenosine triphosphate (high-energy phosphates). At viral level, EOW produces alteration of the capside, DNAse and RNAse enzymes [17,18]; microbicidal properties that suggest having an effect against *T. cruzi*. Therefore, EOW use is a candidate as an innovative treatment against ChD, since there is no drug or product with a curative effect on the market and that does not cause adverse effects as nifurtimox or benznidazole do [19,20].

BALB/c mice were used for the study of ChD for many years by our working group, since it was shown that they share most of the immunological and pathophysiological characteristics of ChD in humans, and they are easy to handle and maintain at low cost, which allows for having a good number of samples that would be a limitation if larger animal models were used [21]. In previous trials, we tested the safety of EOW inoculating it intramuscularly, intravenously, and orally in mice which were followed up for one year, evaluating survival and body condition with positive results, such as 100% survival and excellent body condition similar to healthy control animals (unpublished data).

**Figure 1:**
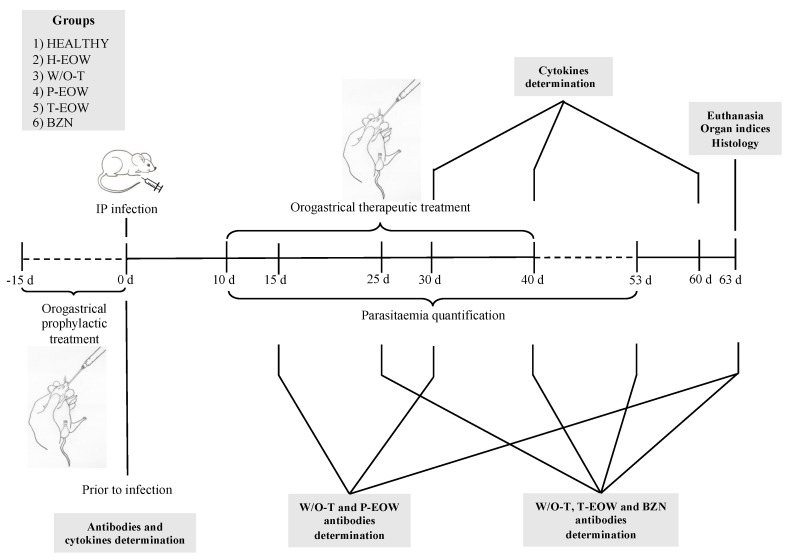
Schematic representation of the methodological design. Six groups of BALB/c mice (*n* = 5) were evaluated: HEALTHY, control noninfected/nontreated; H-EOW, control noninfected and EOW treated; W/O-T, positive control of infection with *T. cruzi* without treatment; P-EOW, infected with *T. cruzi* and treated with prophylactic EOW; T-EOW, infected with *T. cruzi* and treated with therapeutic EOW; and benznidazole (BZN), infected with *T. cruzi* and treated with benznidazole. The infected groups were intraperitoneally infected with 150 blood trypomastigotes of the Ninoa *T. cruzi* strain to evaluate the effectiveness of the prophylactic and therapeutic EOW treatment.

For these reasons, the objective of this study was to evaluate the immunopathological features in *T. cruzi* experimentally infected BALB/c mice treated prophylactically and therapeutically with a pH-neutral EOW. A timeline is included to facilitate tracking of the procedures that were carried out (Figure 1) in this study.

## 2. Results

### 2.1. Beneficial Effects of EOW on Parasitaemia, Health Condition and Survival Rate

**Figure 2:**
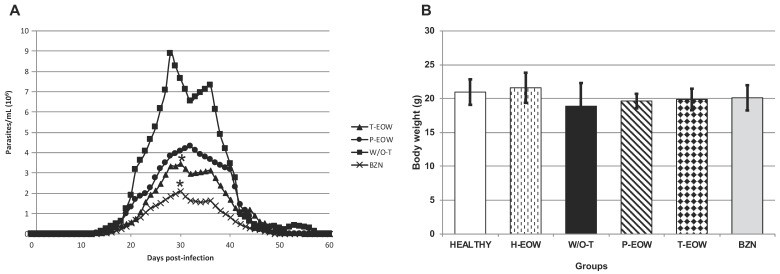

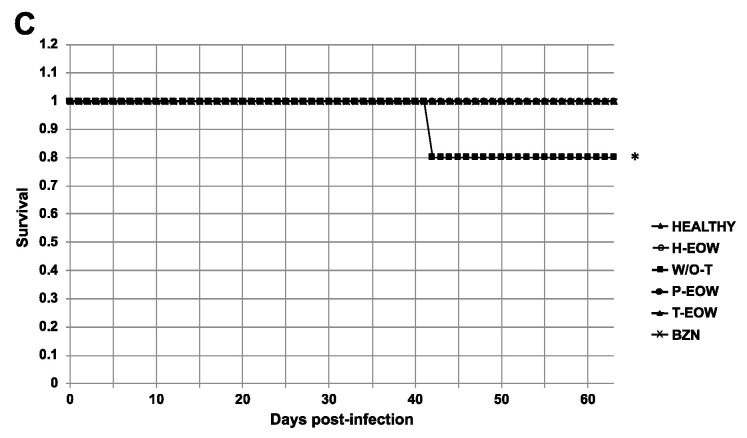
Parasitaemia (**A**), body weight (**B**), and survival rate (**C**) of BALB/c mice experimentally infected with *T. cruzi* and treated with prophylactic or therapeutic EOW. The values plotted on (**A**) show the mean of five mice per group and are representative of two independent experiments. The differences were considered significant (*) at *p* ≤ 0.05 (Kruskal-Wallis test was used, comparing the highest peaks in the treated groups and the one observed in the W/O-T group). The values in (**B**) show the mean ± standard deviation (S.D.). The one-way ANOVA test was used, comparing the data of the different groups vs. the healthy group and vs. the healthy and EOW treated group. The values plotted on (**C**) show the mean ± S.D. of five mice per group and are representative of two independent experiments with similar results. Statistically significant difference (*p* ≤ 0.05) among W/O-T versus the rest of the groups were observed by Kaplan-Meier.

To assess the influence of EOW as experimental treatment on the clinical presentation and lethality of ChD in the acute phase, parasitaemia levels were quantified, clinical follow-up was performed, and daily mortality was recorded. Parasitaemia was detectable in all groups between 13 and 15 days post infection (dpi), ending on days 58 (for the W/O-T group), 56 (for the T-EOW group), 52 (for the P-EOW group) and 51 (for the BZN group). Only one peak of parasitaemia in the P-EOW and BZN groups was observed: 4.3 × 10^6^ parasites/mL of blood (on day 32) in the first group; and 2.08 × 10^6^ parasites/mL of blood (on day 30) in the second one. Two parasitaemia peaks occurred in the W/O-T group (on days 28 and 36), the first being of greater magnitude with 8.87 × 10^6^ parasites/mL of blood. There were three peaks of parasitaemia in the T-EOW group (on days 30, 35 and 44), the first being greater with 3.4 × 10^6^ parasites/mL of blood (Figure 2A). A statistically significant difference was found in the T-EOW (*p* = 0.034) and BZN (*p* = 0.002) groups when comparing their maximum peaks of parasitaemia to that of the W/O-T group; but no statistically significant difference was found between each other (*p* = 1.000). These results demonstrated that therapeutic EOW administration had an effect similar to that of benznidazole treatment on the control of parasites in blood during the acute stage of infection.

During clinical follow-up by examining general physical condition, there were no signs of disease in the four groups that received treatment; while in the W/O-T group signs such as adynamia, arched back and piloerection were observed from 29 dpi. The greatest intergroup discrepancy of body weight was recorded between the W/O-T and the HEALTHY and H-EOW groups, showing differences of 9.9% and 12.6%, respectively (18.88 ± 3.41 g in the W/O-T group vs. 20.96 ± 1.88 g and 21.60 ± 2.22 g in the HEALTHY and H-EOW groups) (Figure 2B). However, no statistically significant differences were found. This suggests that the treatments did not affect the animals’ health status evaluated by physical examination and body condition through weight.

The mice of the W/O-T group showed 80% (4/5) survival rate at the end of the experiment, presenting one death on 42 dpi. Regarding the survival rate in the four groups that received treatment, this was 100% (5/5) at 60 dpi (Figure 2C), which suggests that the treatments were effective in avoiding mortality of mice with acute *T. cruzi* infection, and they did not cause toxicity leading to death (*p* = 0.025).

### 2.2. EOW Treatment Stimulates a Th2 Immune Response in the Early Acute Phase and Th1 in the Late One

**Figure 3:**
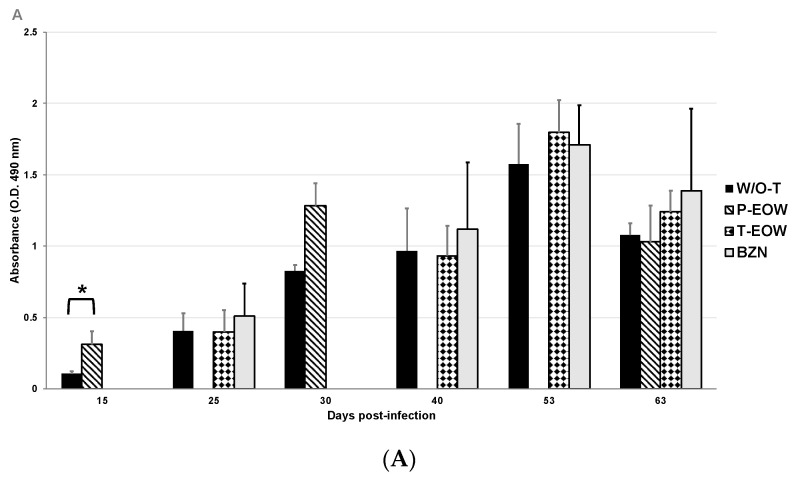

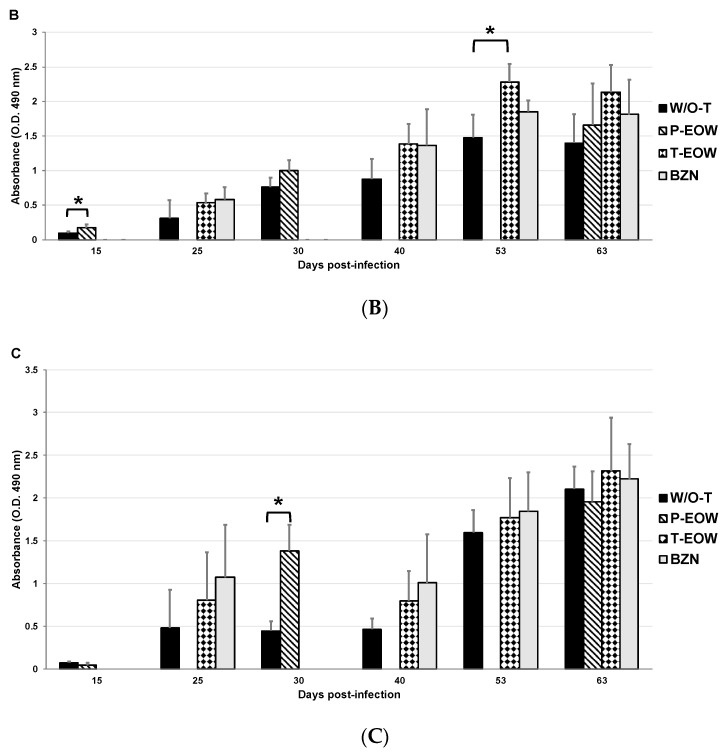
Immunoglobulin levels in sera of BALB/c mice experimentally infected with *T. cruzi* and treated with prophylactic or therapeutic EOW. The values represent the means of duplicate assays ± S.D. for detection of IgG (**A**), IgG1 (**B**) and IgG2a (**C**) titers. The apparent zero values of some groups are determinations that were not carried out at certain timepoints as shown in Figure 1. The differences were considered significant (*) at *p* ≤ 0.05 (Kruskal-Wallis and one-way ANOVA tests were used).

With the serum levels determination of total IgG and its subclasses IgG1 and IgG2a, the humoral immune response induced by the prophylactic and therapeutic EOW administration was demonstrated (Figure 3). Anti-*T. cruzi* IgG production was significantly increased at 15 dpi in the P-EOW group, suggesting a positive effect on the early production of this immunoglobulin (Figure 3A). IgG1 levels in the T-EOW and BZN groups showed a gradual increase during the course of the acute phase, reaching significantly high levels at 53 dpi in the group therapeutically treated with EOW (Figure 3B). With the prophylactic EOW treatment, the production of IgG2a increased significantly from 30 dpi and maintaining high levels until the beginning of the chronic stage (Figure 3C). With these data it is possible to suggest that EOW administration stimulates a Th2 polarized immune response in the early phase of infection, and then toward a Th1-type response at the beginning of the chronic phase of ChD, better than infection alone or than treatment with benznidazole.

### 2.3. Prophylactic EOW Administration Induces a 10-fold Increase of IFN-γ Production in the Acute Phase

**Figure 4:**
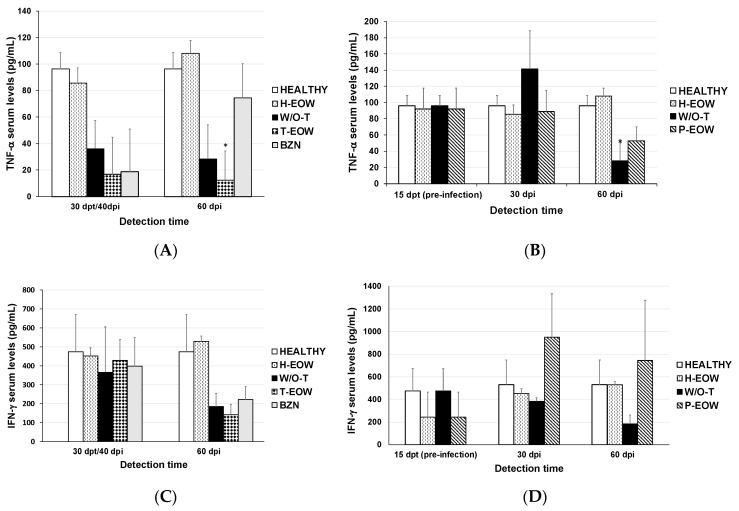

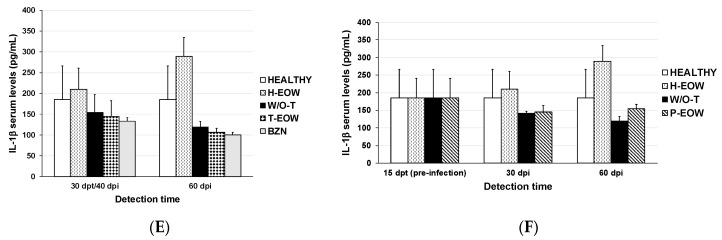
TNF-α, IFN-γ and IL-1β quantification by enzyme-linked immunosorbent assay (ELISA) in sera from *T. cruzi*-infected in BALB/c mice experimentally infected with *T. cruzi* and treated with prophylactic (**B**,**D**,**F**) or therapeutic EOW (**A**,**C**,**E**). The results are shown as group mean ± S.D. The differences were considered significant (*) at *p* ≤ 0.05 when comparing infected and treated groups vs. the healthy control group (Kruskal-Wallis and one-way ANOVA tests were used).

To establish if there was any variation in the cytokine profile expressed during the acute phase of infection in those groups that received experimental treatment, TNF-α, IFN-γ, and IL-1β serum levels were quantified (Figure 4). The cytokine profile of group H-EOW was similar to that of group HEALTHY, so it is inferred that treatment with EOW has no effect on these cytokines evaluated in healthy mice.

TNF-α levels decreased at 30 days post-treatment (dpt) (40 dpi) in the T-EOW, BZN and W/O-T groups compared to the healthy and H-EOW control groups (Figure 4A); however, there was no statistically significant difference. Subsequently, at 60 dpi, the T-EOW group showed a clear statistical difference by further decreasing their TNF-α levels compared to the both healthy control groups (HEALTHY and H-EOW) (*p* = 0.029). In the P-EOW group, the TNF-α production remained at very similar levels at all detection times compared to the healthy control groups in despite of the infection as seen in the W/O-T group, which showed a significant decrease (*p* = 0.038) at 60 dpi. This suggests that to a certain extent, the prophylactic EOW treatment had a positive effect on modulation of the inflammatory response in infected animals in a late acute phase (Figure 4B).

In the therapeutic scheme, IFN-γ levels behaved similarly in all infected groups with or without treatment 30 days after administration (40 dpi); and later, they showed a decrease at 60 dpi; however, there was no statistically significant difference when compared to the levels of the healthy and H-EOW control groups (Figure 4C). In the prophylactic scheme, the P-EOW group showed dramatically low levels of IFN-γ at the end of the preventive treatment (before infection); later, at 30 dpi, its production increased approximately 10-fold until reaching levels close to 1000 pg/mL, which subtly decreased at 60 dpi (Figure 4D). No statistically significant differences were found when the experimental groups were compared to the HEALTHY and H-EOW groups. With these data it is possible to propose that the prophylactic EOW administration contributed to a positive trend in the IFN-γ production during the acute phase, which could be maintained even up to 60 dpi.

IL-1β levels decreased in the groups therapeutically treated with EOW and benznidazole at 40 dpi in the same way as the infected group, and its production continued to decrease until day 60 in all three groups (Figure 4E), without statistical significance. Prophylactic EOW administration showed low IL-1β levels similar to those of W/O-T group at all detection times; however, neither demonstrated significant differences when compared to the HEALTHY and H-EOW control groups; therefore, it is suggested that prophylactic EOW treatment does not have an effect on this cytokine (Figure 4F). 

As expected, the EOW administration in healthy animals does not have an effect on any of the three cytokines since the levels found were very similar to those of the HEALTHY control group; while in infected animals a difference was seen in the levels of some cytokines, thus their immunomodulatory effect is suggested.

### 2.4. Effects of EOW on Cardiomegaly, Splenomegaly and Lymphadenopathy

**Figure 5:**
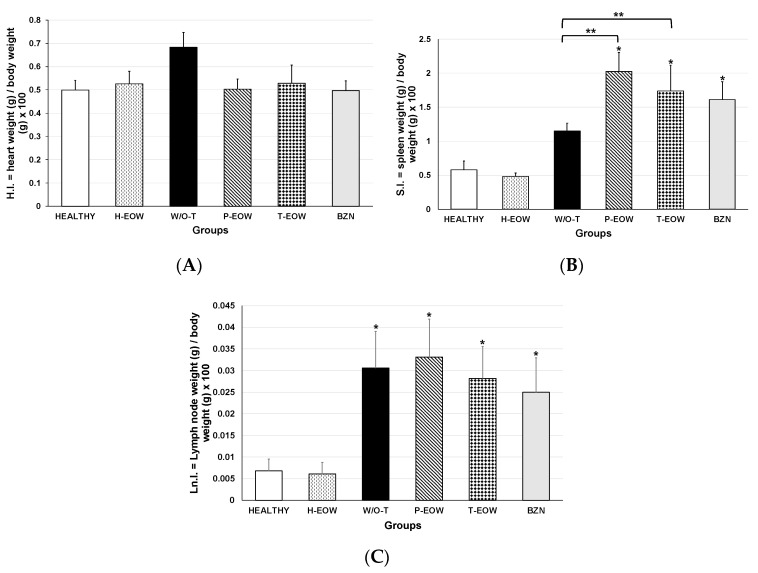
Cardiomegaly, splenomegaly, and lymphadenopathy in BALB/c mice experimentally infected with *T. cruzi* and treated with prophylactic or therapeutic EOW. The values represent the means of the heart index (H.I.), the splenic index (S.I.), and the lymph node index (Ln.I.) of duplicate assays (± S.D.) Cardiomegaly (**A**), splenomegaly (**B**), and lymphadenopathy (**C**) was considered when the organ index was significantly higher than that observed in those from healthy control mice (*) at *p* ≤ 0.05). (**) Significant differences in (B) were also shown when comparing treated groups vs. the infected untreated one. The Kruskal-Wallis and one-way ANOVA tests were used.

To evaluate the effect of the treatment on the development of mega syndromes, the indices of three organs were determined: heart, spleen, and popliteal lymph nodes (Figure 5). Cardiomegaly (Figure 5A) was not observed in any of the experimental groups demonstrated by the average cardiac index compared to the healthy control group (*p* = 0.056). However, a marked trend of increased heart index could be observed in the W/O-T group compared to the healthy control group with a difference of 26.6% (0.68 ± 0.06 in the W/O-T group vs. 0.49 ± 0.04 in the healthy group), which suggested that untreated animals might develop dilated cardiomyopathy or hypertrophy if the disease had progressed to the late chronic stage.

The BZN, P-EOW and T-EOW groups showed splenomegaly (Figure 5B) and lymphadenopathy (Figure 5C) at the end of the course of the acute phase demonstrated by the average splenic and lymph node index, respectively, compared to the healthy control group (*p* = 0.000). This suggested that infected animals, whether treated or not, were responding immunologically to the parasite’s antigenic stimulus in a similar way.

When comparing the splenic index of all the groups that received treatment with that of the W/O-T group, there was also statistically significant differences in the P-EOW (*p* = 0.016) and T-EOW (*p* = 0.001) group; while the splenic index of the BZN group (*p* = 0.141) showed values similar to those of the W/O-T group (Figure 5B). These data indicated that the treatments caused an enlarged spleen, which may suggest that an exacerbation of the immune response may be occurring at a higher level than the infection alone. 

Regarding lymphadenopathy, no statistically significant differences were observed between the peripheral lymph node index of those groups that received treatment compared to the infected untreated group (Figure 5C), demonstrating that even with treatment, the immune response in secondary lymphoid organs showed a behavior with a similar pattern in all infected groups.

The organic indices to determine cardiomegaly, splenomegaly and lymphadenopathy did not show to be different between the uninfected animals (HEALTHY group) and EOW treated non-infected mice (H-EOW group); this demonstrates that oral EOW treatment has no effect on the macroscopic characteristics of these organs.

### 2.5. EOW Administration Ameliorated the Inflammation Degree and Prevented Tissue Parasitism

**Figure 6:**
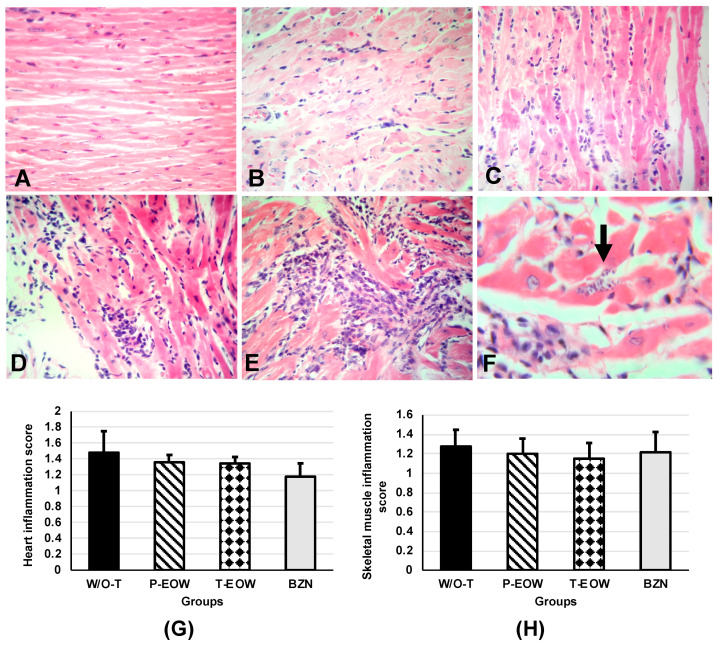
Histological findings from BALB/c mice experimentally infected with *T. cruzi* and treated with prophylactic or therapeutic EOW. Representative micrographs in (**A**–**E**) are shown. (**A**) Score 0 (normal) from a healthy mouse; (**B**) Score 1 (one focus of inflammatory cells/field) from a mouse treated with benznidazole; (**C**) Score 2 (more than one or a few foci of inflammatory cells/field) from a mouse treated prophylactically or therapeutically with EOW; (**D**) Score 3 (generalized coalescent inflammation foci or disseminated inflammation with minimal cell necrosis and preserved tissue integrity) from a mouse treated prophylactically or therapeutically with EOW; (**E**) Score 4 (diffuse inflammation, severe tissue necrosis, interstitial edema, hemorrhage, and/or loss of tissue integrity) from an untreated infected mouse. H&E staining, 40× objective. (**F**) Amastigote nest (arrow) in a heart tissue section from a mouse of the W/O-T group. H&E staining, 100× objective. Inflammatory lesion (inflammatory cell infiltrates) scores of heart (**G**) and skeletal muscle (**H**) are shown. The score was converted into logarithm (base 10) and then 1 was added to each result to correct the values of 0. The results were expressed as group means ± S.D. No differences were observed (Kruskal-Wallis test when comparing the infected and treated groups vs. the infected group without treatment).

With the purpose of evaluating the effect of the experimental treatment on the tissue alterations caused by *T. cruzi*, sections of different typical ChD target organs and others that are indirectly involved were analyzed. A predominantly subepicardial mononuclear inflammatory infiltrate was observed in the myocardial tissue of all infected mice (Figure 6B–E). The inflammation degree in the groups prophylactically and therapeutically treated with EOW had a very similar pattern, being classified with scores between 2 and 3, mostly (Figure 6B–D); the W/O-T group showed very high scores, being classified as the most severe grade (score 4) (Figure 6E), and even amastigote nests were observed in one of the mice (Figure 6F). Inflammation was much lower in the BZN group, as shown by the statistical significance (*p* = 0.027) when compared to the W/O-T group (Figure 6G).

In skeletal muscle, inflammatory infiltrate with the same cellular characteristics as that seen in heart was observed. All groups showed similar inflammation degrees with low scores without statistically significant difference when compared among them (*p* = 0.603) (Figure 6H).

**Figure 7:**
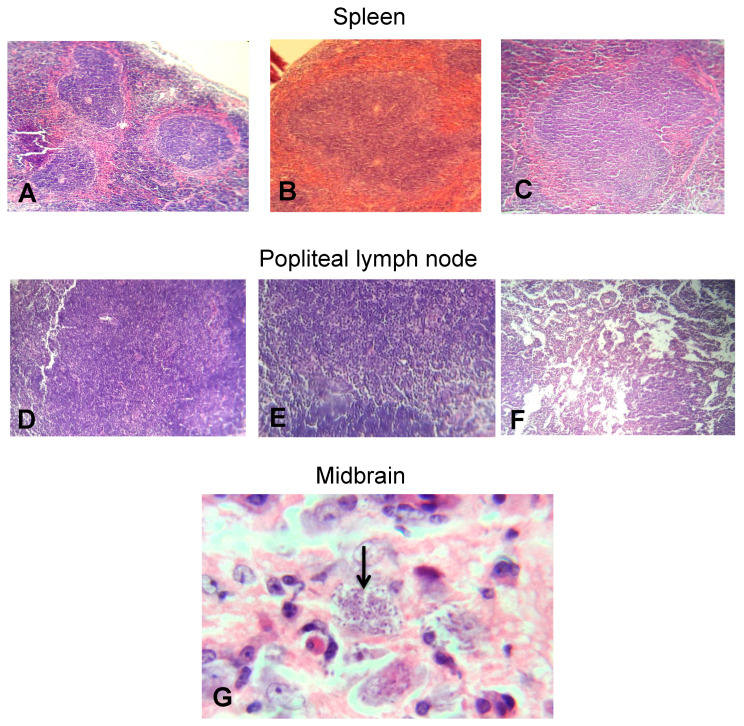
Spleen, popliteal lymph node and midbrain tissue sections from BALB/c mice experimentally infected with *T. cruzi*. (**A**) Spleen of a healthy mouse and (**B**) spleen of a healthy mouse EOW treated, both showing the relationship and normal appearance of the splenic follicles. (**C**) Follicular hyperplasia (+++) in the spleen of a mouse experimentally infected with *T. cruzi* and without treatment. (**D**) Popliteal lymph node from a healthy mouse and (**E**) popliteal lymph node from a healthy mouse EOW treated, both showing normal relationship and appearance. (**F**) Sinusoidal dilation in popliteal lymph node of a mouse experimentally infected with *T. cruzi* and without treatment. H&E staining, 10× objective. (**G**) The presence of multiple parasites (arrow) surrounded by an area of ischemia and vacuolization is observed in a midbrain of a mouse without treatment. H&E staining, 100× objective.

In the W/O-T group, the microscopic findings were not limited to the heart and skeletal muscle, in the majority of the mice splenic follicular hyperplasia was evident (Figure 7C), while in popliteal lymph nodes, sinusoidal dilation was found (Figure 7F). In the midbrain of one of the mice that received no treatment, the parasite load could be corroborated by the presence of trypomastigote forms, which were surrounded by an ischemic zone with vacuolization (Figure 7G). In the gastrointestinal tract (esophagus, small and large intestine), no microscopic changes were observed in any of the groups (data not shown). These findings suggested that both prophylactic and therapeutic EOW administration contributed to the partial control of inflammation and the total control of tissue parasitism, results that resemble those found in the group of mice treated with benznidazole. The histological sections of healthy mice treated with EOW showed no apparent pathological features, which proves its safety.

## 3. Discussion

A variety of electrolyzed waters has been widely used in the food industry, and in dental, medical, and veterinary processes against a large number of microorganisms such as bacteria, fungi, viruses, and spores. This study is innovative as the effect of one type of electrolyzed water against a protozoal disease is explored for the first time. In the present study the therapeutic and prophylactic effect of an EOW on the modulation of the humoral and cellular immune response was evaluated in the murine model of ChD. Both prophylactic and therapeutic EOW treatments decreased parasitaemia, improved the clinical course, reduced mortality and were able to control the tissue parasitism compared to the group without treatment; and even with better results than those found in the BZN group.

The safety of the electrolyzed functional water was already demonstrated by Morita et al. (2011); they observed that mice that drink acid electrolyzed functional water ad libitum for eight weeks showed no significant differences in changes in body weight, condition of coat, condition of feces, and overall visual findings compared to the control group. They also found no abnormalities in terms of visual inspections of the oral cavity, histopathological tests, or tooth surface enamel roughness measurements. They only observed enamel attrition in some molars in the test group with the electrolyzed water. This research group suggested the use of acid pH electrolyzed water does not have a systemic effect and is safe for mouthwash [22].

Electrolyzed reducing water (ERW) was shown to protect the cellular redox balance, reducing the risk of various diseases with altered cellular homeostasis such as inflammation [23]. Several biological results of ERW were reported, such as protective effect of oxidative damage to DNA by reactive oxygen species [24], stimulator of growth of anaerobic microflora in the human gut [25], antidiabetic effect in rats and mice [26,27], antioxidant effect [28], growth-promoting effect of the fetus in rats [29], therapeutic effect in patients with end-stage renal disease [30] and reduction in blood viscosity due to dehydration in humans [31].

Currently, there are multiple studies that describe the interaction among different protective mechanisms of the immune system in response to *T. cruzi* infection. These mechanisms involve different cell lines activation, pro-inflammatory cytokines (TNF-α and IL-1β) and Th1-type (IFN-γ and IL-12) production, and increased reactive oxygen species production [32]. Although these elements favor the control of parasitism in the acute phase, their overexpression during the chronic phase is the cause of cardiac remodeling and progression to chronic chagasic cardiomyopathy, a clear example of this is the dual role of IFN-γ in different phases of ChD [33].

Among the objectives of this research was to determine the effect of EOW treatment on the humoral immune response in mice infected with *T. cruzi*. Although extensive research was done with the use of EOW, the effect on antibody production was not previously explored in any animal model. In this regard, the total IgG levels and the IgG1 and IgG2a subclasses were quantified. Our results indicated that the treatment induced IgG2a antibodies related to a Th1-type response, which correlates with the profile of cytokines found. 

It was shown that several recombinant *T. cruzi* proteins can generate Th1-type antibodies in vaccinated mice and this kind of antibodies is required to eliminate the parasite in infected animals [34,35]. Although an immunogen was not used in the present study, the EOW had an immunomodulatory effect similar to that of recombinant vaccines, based on IgG2a subclass antibodies predominantly produced in infected animals prophylactically treated with EOW.

On the other hand, the mice treated prophylactically with EOW had a lower amount of IL-1β and TNF-α and higher levels of IFN-γ than untreated animals. Something similar was found in other studies, which demonstrate the useful of electrolyzed water as treatment in two types of conditions in mouse model; Lee et al. (2009) showed that electrolyzed reduced water decreased the production of IL-1β and TNF-α in C57BL/6 mice infected with *Echinostoma hortense* (intestinal helminth) metacercariae [36]. Low levels of intestinal TNF-α expression were similar in mice that given free access to tap water as drinking water to those that were given free access to acid electrolyzed functional water as drinking water [22]. More recently, You’s group (2017) observed that treatment with slightly electrolyzed acidic water in cutaneous wounds in hairless mice decreased serum proinflammatory cytokines such as IL-1β, IL-6, keratinocyte chemoattractant and TNF-α [37]. 

The IFN-γ peak in the P-EOW group at 30 dpi could not be directly correlated with a beneficial effect on other parameters determined in this study, since this group expressed characteristics very similar to those treated therapeutically with EOW, and even those of the BZN group, which had less histological damage and better control of parasitaemia even without this IFN-γ overexpression. The foregoing does not agree with what was found by others who studied the effects of benznidazole on the immune system, since it was described that there is a synergistic action of this drug when it is administered in the acute phase together with IL-12, which in turn triggers the IFN-γ overproduction [38]. While being administered in the chronic phase, it is capable of modulating IFN-γ levels and reducing its production [39].

The cytokines’ expression determined in this study also differs from that described by other authors who used the same *T. cruzi* strain (MHOM/MX/1994/Ninoa) and described how these cytokines production is triggered during the first half of the acute phase during the natural course of *T. cruzi* infection, and then gradually decreases. Otherwise, the clinical course of the infection observed here, as well as the low levels of parasitaemia and mortality do agree with those reported by others, despite the use of different infection inoculums [40].

The absence of cardiomegaly and the presence of splenomegaly and lymphadenopathy were constant in most of the experimental groups evaluated. These results are consistent with those found in other animal models [41], in which it is described that while the acute phase is characterized by this type of findings, in the chronic phase a slight splenomegaly, absence of lymphadenopathy and a marked cardiomegaly predominate. Therefore, the splenic and secondary lymphatic organs responses in the late acute phase of the infection were similar in all groups treated with EOW and with benznidazole. However, while in the W/O-T group it was not possible to show the splenomegaly, there were histological changes that showed an inflammatory response, resulting in reactive lymphoid hyperplasia.

On the other hand, the presence of a diffuse mononuclear inflammatory infiltrate in the heart and skeletal muscle was constant, which is consistent with the findings already described by different groups in other animal models during the acute phase, and even in human samples [42]. In this regard, the results obtained in the present study showed that the W/O-T group had the highest inflammation severity scores in heart tissue, and it was possible to identify the parasite’s presence in this organ and in the central nervous system. However, cytokine production was found in low ranges and similar to the other treated groups, which differs from that described in the literature, where it is established that the exaggerated increase in the inflammatory infiltrate characteristic of ChD in myocardial tissue correlates with increased production of cytokines such as IFN-γ during the acute phase [33,41] and other proinflammatory ones. It is suggested that the immunomodulation observed in the other treated groups was associated with the antigenic stimulus of *T. cruzi* infection and not with the administration of EOW alone by showing a very similar cytokine profile between HEALTHY and H-EOW groups.

In recent years, the study of the polarization between the Th1- and Th2-type responses was addressed, which are closely related by mutually inhibiting each other, and it was observed that the Th1-type cytokines decrease makes the host more susceptible to infection [43]. In this study, Th2-type cytokines were not determined based on the determination of IgG1 subclass to evaluate the TH2 immune response, so we cannot confirm whether the low levels of TNF-α, IFN-γ and IL-1β were due to an overproduction of other antagonists such as IL-10 and IL-4. However, if the increase in the latter were present, this would be paradoxical with what was described by our research group previously [44], in which high mortalities in experimental animals when the immune response is polarized toward Th2 were reported; this did not happen in the present study, where the clinical course was favorable in the groups treated with EOW and benznidazole. This could suggest two possible scenarios: that the infection control was elicited by the direct toxic effect of the administered products on the parasite, or that there could be synergy with another type of immune response such as humoral or complement through other signaling pathways unexplored, placing EOW as an immunomodulatory agent. Regarding the preferred subclass of antibodies generated (IgG2a) and cytokine profile (Th1) induced, it is possible to postulate that EOW leads to immunological modulation that greatly ameliorated *T. cruzi* infection. This agrees with other authors’ reports, which demonstrate that electrolyzed solutions helped to increase the body’s immune defenses against oxidative stress by eliminating in particular hydrogen peroxide and the hypochlorite anion, as well as improved the inflammatory response and the control of blood pressure in hemodialysis patients [45]. Kapur and Marwaha (2011) showed that there was remarkable reduction in common signs of inflammation such as edema, erythema, and drastic increase of granulation and fibrin formation in patients with different types of wounds treated with EOW demonstrating an immunomodulatory effect [46]. In this regard, immunotherapy with the EOW could provide a survival advantage by reducing the clinical signs of infection and ameliorating the cardiac damage of Chagas disease by avoiding disease progression as seen with other immunotherapeutic agents against various pathologies, such as allergies, injury to the peripheral nervous system, Chagas disease, leishmaniosis, and other intracellular pathogens [47,48,49,50,51]. In this study, high parasitaemia, general physical condition (adynamia, arched back and piloerection), lethality and tissue parasite load considered to be typical clinical manifestations of ChD were ameliorated in mice treated with EOW. 

New questions arise about our findings, which could be answered by complementing this study with the determination of a more extensive panel of Th1-and Th2-type cytokines, or lymphoproliferation tests; and the search for lytic antibodies mediated by the action of the complement. It is also proposed to extend the study to later stages of the chronic phase of ChD in order to obtain more data that allows us to define the effect of EOW on the pathogenesis of the disease more precisely. 

## 4. Materials and Methods

### 4.1. Animals and Experimental Groups

Sixty 6 to 8-week old female BALB/c mice (from the Center for Research and Advanced Studies of the National Polytechnic Institute CINVESTAV-IPN, for the acronym in Spanish) were divided into six groups with five animals per group: (1) healthy mice without infection or treatment (HEALTHY), (2) healthy mice without infection treated with EOW (H-EOW), (3) infected mice without treatment (W/O-T), (4) infected mice prophylactically treated with EOW (P-EOW), (5) infected mice therapeutically treated with EOW (T-EOW) and 6) infected mice treated with benznidazole (BZN). Experiments were carried out in duplicate to confirm reproducibility. All mice were housed with light/dark cycles of 12 h/12 h, under temperature conditions of 22–24 °C, with food and water ad libitum. All procedures were performed under the protocols established by the Guide for Care and Use of Experimental Animals, National Institutes of Health [52] and the official Mexican norm (NOM-062-ZOO-1999) about Technical Specifications for the Production, Care and Use of Laboratory Animals [53], with approval by the Internal Committee for the Care and Use of Laboratory Animals (CICUAL, for the acronym in Spanish) registered under the number INC/CICUAL/001/2017.

### 4.2. Infection

Mice were inoculated with blood trypomastigotes (BT) of the Ninoa *T. cruzi* strain (MHOM/MX/1994/Ninoa (*T. cruzi*)). The parasites were obtained from blood samples from the tail vein of previously infected mice (approximately at 21 days after infection). The parasites were counted in a Neubauer chamber and the volume was adjusted to infect each mouse with 150 BT intraperitoneally, using sterile syringes with a 27 G × 13 mm needle. 

### 4.3. Parasitaemia and Survival

Parasitaemia curves were elaborated by parasite counts per blood sample every third day from 10 dpi and until the blood sample was negative for the presence of parasitic forms by the modified Petana method [54] as follows: 10 µL of blood was obtained from the tail vein; they were diluted in 490 µL of physiological 0.9% saline solution (SS) to obtain a 1:50 dilution. The Neubauer chamber was loaded with 10 µL of this dilution, and BT was counted in the four squares used for leukocyte counting using 25× and 40× objectives. The total number of parasites counted was multiplied by 50 (dilution factor), divided by 0.4 resulting from the product of the chamber surface (4 mm^2^) and its depth (0.1 mm); finally, this result was multiplied by 1000 to obtain the number of parasites in 1 mL of blood. Also, survival was recorded daily and the mortality rate (number of mice that died during acute infection) was obtained.

### 4.4. Prophylactic EOW and Therapeutic EOW Treatments by Orogastric Route

The pH-neutral EOW used in this study was SoluVet^®^, a commercial product provided by Esteripharma México, S.A. de C.V. SoluVet^®^ is an EOW with neutral pH at 0.0006% (60 parts per million, ppm) of free active chlorine. For the prophylactic treatment, 500 µL of EOW was administered every 24 h for 15 days prior to infection, and for the therapeutic treatment the same amount of EOW every 24 h for 30 days, starting on the tenth day after infection; both by orogastric route. The H-EOW control group was treated under the same scheme of administration as the T-EOW group. As a positive control, an active ingredient obtained from 100 mg benznidazole LaFepe^®^ (Laboratório Farmacêutico do Estado de Pernambuco, São José Recife, Brazil) tablets was used preparing a suspension with Tween 20 at 5%, adjusting the dose of 10 mg/kg to the volume used per dose (500 µL); this dose was administered every 24 h for 30 days by orogastric route, starting on the tenth day after infection. The treatments were performed with a No. 18 straight stainless-steel feeding cannula (Cadence Science Inc.^®^, Pike, Cranston, RI, USA).

### 4.5. Collection, Handling and Storage of Blood Samples

A 250–300 µL blood sample was taken from each mouse prior to the experiments to be used as a reference (preimmune serum). For the T-EOW, BZN and W/O-T groups, samples were collected twice: at 40 dpi, equivalent to 30 dpt, and 60 dpi. In the P-EOW group, samples were collected three times: on day 15 after the start of preventive treatment (preinfection), and at 30 and 60 dpi; and in the H-EOW group, samples were collected on days 15, 30 and 60 dpt. Likewise, an additional blood sample was extracted from the group without treatment at 30 dpi. The blood collection was carried out at 3 h post-treatment from the tail vein and collected in 1.5 mL tubes for microcentrifuge without anticoagulant, kept at room temperature until clot retraction and then centrifuged at 3500 rpm for 15 min at a temperature of 4 °C using a refrigerated microcentrifuge (model RMC-14, Sorvall^®^/DuPont^®^). Finally, aliquots of 50–100 μL of sera were made in 0.6 mL tubes for microcentrifuge and stored in freezing at −20 °C until use.

### 4.6. Immunoglobulins Determination

Total IgG antibody and subclasses IgG1 and IgG2a, were evaluated at 15, 25 (at the middle of the therapeutic EOW and benznidazole administrations), 30, 40 (at the end of the therapeutic EOW and benznidazole administration), 53 and 63 dpi by the enzyme-linked immunosorbent assay (ELISA) method according to the manufacturer’s instructions (Novus Biologicals, Centennial, CO, USA). Briefly, plates were coated with the whole *T. cruzi* INC-9 isolate extract (1 μg/mL) in 200 μL of carbonate buffer (NaCO_3_/NaHCO_3_ pH 9.6) and incubated for 1 h at 37 °C. Plates were washed five times with PBS 1×-0.05% Tween-20 (PBS-T), incubated for 30 min at 37 °C with a blocking solution (PBS containing 0.5% bovine serum albumin fraction V). Serum samples were diluted in blocking buffer at dilution of 1:200 in 200 μL/well and incubated (1 h, 37 °C). As a negative control, a pool of preimmune sera was used in all experiments. The plates were washed five times with PBS-T. After washing, the peroxidase-labeled rabbit anti-mouse IgG and IgG subclasses (IgG1 and IgG2a) secondary antibodies (Novus Biologicals, Centennial, CO, USA) were added at 1:10,000 dilution in PBS-T and incubated for 1 h at room temperature. The plates were washed five times with PBS-T, 150 μL of peroxidase substrate OPD (*orto*-phenylenediamine dihydrochloride, Sigma Aldrich, St. Louis, MO, USA) in citrate buffer at pH 4.5–0.03% H_2_O_2_ was added, and incubated for 10 min at room temperature. The reaction was stopped 10 min later by the addition of 50 μL of 5 N H_2_SO_4_. Absorbance values were determined at 495 nm in a Microplate Reader (Bio-Rad, Hercules, CA, USA). All measurements were performed twice.

### 4.7. Cytokine Determination

Th1 cytokine levels (TNF-α, IFN-γ, and IL-1β,) in the mice’s serum at 15 dpt (preinfection in the prophylactic EOW treatment), 40 dpi (at the end of the therapeutic EOW and benznidazole administration) and 60 dpi were measured by duplicate by ELISA using commercial kits (Enzo Life Sciences, Inc.^®^, East Farmingdale, NY, USA and R&D Systems, Inc.^®^, Minneapolis, MN, USA) according to the manufacturer’s instructions. Briefly, the capture antibody was diluted to 1.0 μg/mL with PBS, 50 μL/well was added to the 96 Maxisorb plates (R&D Systems Inc.^®^, Minneapolis, MN, USA) and incubated overnight at room temperature. The plates were washed three times with 250 μL PBS-T, blocked with 200 μL of blocking buffer per well for 1 h at room temperature and washed four times. The standard sample was prepared for each cytokine and 50 μL/well was added; then, 50 μL of the serum samples were added to each well and incubated for 2 h at room temperature. The plates were washed three times, and then 50 μL of the previously diluted detection antibody (1.5 μg/mL) was added and incubated at room temperature for 2 h. The plates were washed, and then 50 μL of previously diluted avidin were added and incubated for 20 min at room temperature. The plates were washed again, and then 50 μL of peroxidase substrate TMB- H_2_O_2_ (3,3’,5,5’-Tetramethylbenzidine, Sigma Aldrich, St. Louis, MO, USA, 30% hydrogen peroxide) was added. The plates were incubated for 20 min at room temperature, and the reaction was stopped with 50 μL of 5N H_2_SO_4_ per well. The reading was performed at 450 nm using a Microplate Reader (model Opsys MR, Dynex Technologies^®^, Chantilly, VA, USA). Each cytokine concentration was determined on the basis of each corresponding standard curve and expressed in pg/mL. 

### 4.8. Splenomegaly, Cardiomegaly and Lymphadenopathy

Prior to euthanasia (at 60 dpi), the body weight of each mouse was obtained, and the weights of the heart, the spleen, and the peripheral lymph nodes (poplitei) were collected during necropsy and were weighed. The organ indices were calculated by applying the following formula: organ weight/body weight ×100. The presence of cardiomegaly, splenomegaly and/or lymphadenopathy was considered when the calculated organ index was significantly higher than that observed in those from healthy uninfected control animals [41].

### 4.9. Histology

The animals were subjected to euthanasia at 60 dpi to aseptically remove heart, intestines (ileum and colon sections), popliteal lymph nodes, skeletal muscle, esophagus, spleen, and brain. The organs were rinsed with SS, fixed in 10% formaldehyde (pH 7.4) and kept at room temperature until their processing. Samples were dehydrated with absolute ethanol, rinsed with xylene, and embedded in paraffin. Sections of 5 µm were made, stained with hematoxylin and eosin and evaluated by optical microscopy (Carl Zeiss^®^ K7, Oberkochen, BW, Germany). The walls of cardiac tissue were analyzed from the upper, middle, and lower parts: subepicardium, myocardium, and subendocardium. The severity of inflammation was scored on a scale of 0 to 4, where 0 = no alterations; 1 = one focus of inflammatory cells/field (400×); 2 = more than one focus of inflammatory cells/field; 3 = generalized coalescing foci of inflammation or disseminated inflammation with minimal cell necrosis and retention of tissue integrity; and 4 = diffuse inflammation, tissue necrosis, interstitial edema, hemorrhage and loss of tissue integrity [55].

### 4.10. Statistical Analysis

All results were analyzed using the IBM^®^ SPSS software (v. 20.0.0) (Armonk, NY, USA). Data were expressed as means ± standard deviation (S.D.) of each group. Prior to the comparison between groups and possible interactions between them, all the variables were analyzed by the Shapiro-Wilk test considering those with a value of *p* ≥ 0.05 as normal distribution. Data with a normal distribution were analyzed by the one-way ANOVA test, followed by Tukey analysis as a post-*hoc* test. Those that did not fit into a normal distribution were analyzed by the Kruskal-Wallis test. The survival analysis was performed using the Kaplan-Meier method. All differences were considered significant when *p* ≤ 0.05. The inflammation grade score was converted into logarithms (base 10) and then 1 was added to each result to correct the values of 0 [56].

## 5. Conclusions

Therapeutic EOW administration was useful for the control of the acute phase of ChD by observing parameters of parasitaemia, mortality, clinical status, histological damage, and production of IgG-type antibodies similar to those obtained with benznidazole treatment. However, it was not possible to correlate these results with a synergy in the cellular-type immune response when determining the levels of the three cytokines. The efficacy observed by EOW could be due to immunomodulatory properties and not to a toxic effect on the parasite.

## Abbreviations

BZN, benznidazole treatment; ChD, Chagas disease; CICUAL, Comité para el Cuidado y Uso de los Animales de Laboratorio; CINVESTAV-IPN, Centro de Investigación y de Estudios Avanzados del Instituto Politécnico Nacional; COFEPRIS, Comisión Federal para la Protección contra los Riesgos Sanitarios; dpi, days postinfection; dpt, days post-treatment; ELISA, enzyme-linked immunosorbent assay; EOW, electrolyzed oxidizing water; IFN-gamma, interferon gamma; IgG, immunoglobulin G; IL-1 beta, interleukin 1 beta; P-EOW, prophylactic electrolyzed oxidizing water treatment; ERW, electrolyzed reducing water; SS saline solution; T-EOW, therapeutic electrolyzed oxidizing water treatment; TNF-alpha, tumor necrosis factor alpha; USD, United States dollar; WHO, World Health Organization; W/O-T, without treatment.

## References

[1] World Health Organization (WHO) (2018). Chagas Disease (American Trypanosomiasis). http://www.who.int/mediacentre/factsheets/fs340/en/.

[2] Ramsey J.M., Elizondo-Cano M., Sanchez-González G., Peña-Nieves A., Figueroa-Lara A. (2014). Opportunity Cost for Early Treatment of Chagas Disease in Mexico. PLoS Negl. Trop. Dis..

[3] Molina I., Salvador F., Sánchez-Montalvá A. (2016). Actualización en enfermedad de Chagas. Enferm. Infecc. Microbiol. Clin..

[4] Salvador F., Treviño B., Sulleiro E., Sánchez-Montalva A., Cabezos J., Soriano A., Serre N., Gómez i Prat J., Pahissa A., Molina I. (2014). *Trypanosoma cruzi* infection in a non-endemic country: Epidemiological and clinical profile. Clin. Microbiol. Infect..

[5] Carabarin-Lima A., González-Vázquez M.C., Rodríguez-Morales O., Baylón-Pacheco L., Rosales-Encina J.L., Reyes-López P.A., Arce-Fonseca M. (2013). Chagas disease (American tripanosomiasis) in Mexico: An update. Acta Trop..

[6] Arce-Fonseca M., Ballinas-Verdugo M.A., Abreu Zenteno E.R., Suárez-Flores D., Carrillo-Sánchez S.C., Alejandre-Aguilar R., Rosales-Encina J.L., Reyes P.A., Rodríguez-Morales O. (2013). Specific humoral and cellular immunity induced by *Trypanosoma cruzi* DNA immunization in a canine model. Vet. Res..

[7] Teixeira A.R.L., Hecht M.M., Guimaro M.C., Sousa A.O., Nitz N. (2011). Pathogenesis of Chagas’ Disease: Parasite Persistence and Autoinmunity. Clin. Microbiol. Rev..

[8] Cardillo F., Teixeira P.R., Zuquim A.P.R., Mengel J. (2015). Immunity and immune modulation in *Trypanosoma cruzi* infection. Pathog. Dis..

[9] Basso B. (2013). Modulation of immune response in experimental Chagas disease. World J. Exp. Med..

[10] Geiger A., Bossard G., Sereno D., Pissarra J., Lemesre J.-P., Vincendeau P., Holzmuller P. (2016). Escaping Deleterious Immune Response in Their Hosts: Lessons from Trypanosomatids. Front. Immunol..

[11] Cardoso M.S., Reis-Cunha J.L., Bartholomeu D.C. (2016). Evasion of the Immune Response by *Trypanosoma cruzi* during Acute Infection. Front. Immunol..

[12] Rodríguez-Morales O., Monteón-Padilla V., Carrillo-Sánchez S.C., Rios-Castro M., Martínez-Cruz M., Carabarin-Lima A., Arce-Fonseca M. (2015). Experimental Vaccines against Chagas Disease: A Journey through History. J. Immunol. Res..

[13] Bern C. (2015). Chagas’ Disease. N. Engl. J. Med..

[14] Caldas S., Caldas I.S., Cecílio A.B., Diniz L.D., Talvani A., Ribeiro I., Bahia M.T. (2014). Therapeutic responses to different anti-*Trypanosoma cruzi* drugs in experimental infection by benznidazole-resistant parasite stock. Parasitology.

[15] Manne J.M., Snively C.S., Ramsey J.M., Salgado M.O., Bärnighausen T., Reich M.R. (2013). Barriers to Treatment Access for Chagas Disease in Mexico. PLoS Negl. Trop. Dis..

[16] Nachón-García F.J., Díaz-Tellez J., Nachón-García M.G. (2005). Tolerancia peritoneal a la solución de alta selectividad iónica con pH neutro en ratas macho Wistar. Rev. Med. Univ. Veracruzana.

[17] Cabello G.C., Rosete O.D.P., Manjarrez Z.M.E. (2009). Efecto de una solución electrolizada de superoxidación con pH neutro sobre la infección del virus de influenza A en células MDCK. Rev. Inst. Nal. Enf. Resp..

[18] García F.J.N., Téllez J.D., Espinoza V.R., González J.S., García M.N., García F.G., García J.S. (2008). Esterilización por inmersión. Estudio comparativo entre glutaraldehído al 2%, agua electrolizada superoxidada con pH neutro y solución electrolizada por selectividad iónica con pH neutro. Rev. Med. Univ. Veracruzana.

[19] Rassi A., Rassi A., de Rezende J.M. (2012). American trypanosomiasis (Chagas disease). Infect. Dis. Clin..

[20] Pérez-Molina J.A., Molina I. (2018). Chagas disease. Lancet.

[21] Jelicks L.A., Tanowitz H.B. (2011). Advances in Imaging of Animal Models of Chagas Disease. Adv. Parasitol..

[22] Morita C., Nishida T., Ito K. (2011). Biological toxicity of acid electrolyzed functional water: Effect of oral administration on mouse digestive tract and changes in body weight. Arch. Oral. Biol..

[23] Franceschelli S., Gatta D.M., Pesce M., Ferrone A., Patruno A., de Lutiis M.A., Grilli A., Felaco M., Croce F., Speranza L. (2016). New Approach in Translational Medicine: Effects of Electrolyzed Reduced Water (ERW) on NF-κB/iNOS Pathway in U937 Cell Line under Altered Redox State. Int. J. Mol. Sci..

[24] Shirahata S., Kabayama S., Nakano M., Miura T., Kusumoto K., Gotoh M., Hayashi H., Otsuo K., Morisawa S., Katakura Y. (1997). Electrolyzed-reduced water scavenges active oxygen species and protects DNA from oxidative damage. Biochem. Biophys. Res. Commun..

[25] Vorobjeva N.V. (2005). Selective stimulation of the growth of anaerobic microflora in the human intestinal tract by electrolyzed reducing water. Med. Hypotheses.

[26] Jin D., Ryu S.H., Kim H.W., Yang E.J., Lim S.J., Ryang Y.S., Chung C.H., Park S.K., Lee K.J. (2006). Anti-diabetic effect of alkaline-reduced water on OLETF rats. Biosci. Biotechnol. Biochem..

[27] Kim M.J., Kim H.K. (2006). Anti-diabetic effects of electrolyzed reduced water in streptozotocin-induced and genetic diabetic mice. Life Sci..

[28] Hanaoka K., Sun D., Lawrence R., Kamitani Y., Fernandes G. (2004). The mechanism of the enhanced antioxidant effects against superoxide anion radicals of reduced water produced by electrolysis. Biophys. Chem..

[29] Watanabe T. (1995). Effect of alkaline ionized water on reproduction in gestational and lactational rats. J. Toxicol. Sci..

[30] Huang K.C., Yang C.C., Lee K.T., Chien C.T. (2003). Reduced hemodialysis-induced oxidative stress in end-stage renal disease patients by electrolyzed reduced water. Kidney Int..

[31] Weidman J., Holsworth R.E., Brossman B., Cho D.J., Cyr J.S., Fridman G. (2016). Effect of electrolyzed high-pH alkaline water on blood viscosity in healthy adults. J. Int. Soc. Sports Nutr..

[32] Machado F.S., Dutra W.O., Esper L., Gollob K.J., Teixeira M.M., Factor S.M., Weiss L.M., Nagajyouthi F., Tanowithz H.B., Garg N.J. (2012). Current Understanding of Immunity to *Trypanosoma cruzi* infection and Pathogenesis of Chagas Disease. Semin. Immunopathol..

[33] Gomes J.A.S., Bahia-Oliveira L.M.G., Rocha M.O.C., Martins-Filho O.A., Gazzinelli G., Correa-Oliveira R. (2003). Evidence that Development of Severe Cardiomyopathy in Human Chagas’ Disease Is Due to a Th1-Specific Immune Response. Infect. Immun..

[34] Hoft D.F., Eickhoff C.S. (2002). Type 1 immunity provides optimal protection against both mucosal and systemic *Trypanosoma cruzi* challenges. Infect. Immun..

[35] Paiva C.N., Castelo-Branco M.T., Lannes-Vieira J., Gattass C.R. (1999). *Trypanosoma cruzi*: Protective response of vaccinated mice is mediated by CD8+ cells, prevents signs of polycolonal T lymphocyte activation, and allows restoration of a resting immune state after challenge. Exp. Parasitol..

[36] Lee K.J., Jin D., Chang B.S., Teng Y.C., Kim D.H. (2009). The immunological effects of electrolyzed reduced water on the *Echinostoma hortense* infection in C57BL/6 mice. Biol. Pharm. Bull..

[37] You H.S., Fadriquela A., Sajo M.E.J., Bajgai J., Ara J., Kim C.S., Kim S.K., Oh J.R., Shim K.Y., Lim H.K. (2017). Wound Healing Effect of Slightly Acidic Electrolyzed Water on Cutaneous Wounds in Hairless Mice via Immune-Redox Modulation. Biol. Pharm. Bull..

[38] Michailowsky V., Mursta S.M.F., Carvalho-Oliveira L., Pereira M.E., Ferreira L.R., Brener Z., Romanha A.J., Gazzinelli R.T. (1998). Interleukin-12 Enhances In Vivo Parasiticidal Effect of Benznidazole during Acute Experimental Infection with a Naturally Drug-Resistant Strain of *Trypanosoma cruzi*. Antimicrob. Agents Chemother..

[39] Cutrullis R.A., Moscatelli G.F., Moroni S., Volta B.J., Cardoni R.L., Altcheh J.M., Corral R.S., Freilij H.L., Petray P.B. (2011). Benznidazole Therapy Modulates Interferon-γ and M2 Muscarinic Receptor Autoantibody Responses in *Trypanosoma cruzi*-Infected Children. PLoS ONE.

[40] Espinoza B., Rico T., Sosa S., Oaxaca E., Vizcaino-Castillo A., Caballero M.L., Martínez I. (2010). Mexican *Trypanosoma cruzi T. cruzi* I Strains with Different Degrees of Virulence Induce Diverse Humoral and Cellular Immune Responses in a Murine. J. Biomed. Biotechnol..

[41] Guedes P.M.M., Veloso V.M., Afonso L.C.C., Caliari M.V., Carneiro C.M., Diniz L.F., Marques-da-Silva E.A., Caldas I.S., Do Valle M.M.A., Souza S.M. (2009). Development of chronic cardiomyopathy in canine Chagas disease correlates with high IFN-γ, TNF-α, and low IL-10 production during the acute infection phase. Vet. Immunol. Immunopathol..

[42] Parada H., Carrasco H.A., Añez N., Fuenmayor C., Inglessis I. (1997). Cardiac involvement is a constant finding in acute Chagas’ disease: A clinical, parasitological and histopathological study. Int. J. Cardiol..

[43] Gupta S., Garg N.J. (2010). Prophylactic Efficacy of TcVac2 against *Trypanosoma cruzi* in Mice. PloS Negl. Trop. Dis..

[44] Arce-Fonseca M., Ramos-Ligonio A., López-Monteón A., Salgado-Jiménez B., Talamás-Rohana P., Rosales-Encina J.L. (2011). A DNA Vaccine Encoding for *Tc*SSP4 Induces Protection against Acute and Chronic Infection in Experimental Chagas Disease. Int. J. Biol. Sci..

[45] Henry M., Chambron J. (2013). Physico-Chemical, Biological and Therapeutic Characteristics of Electrolyzed Reduced Alkaline Water (ERAW). Water.

[46] Kapur V., Marwaha A.K. (2011). Evaluation of effect and comparison of superoxidised solution (oxum) v/s povidone iodine (betadine). Indian J. Surg..

[47] Ezz-Eldin Y.M., Aboseif A.A., Khalaf M.M. (2020). Potential anti-inflammatory and immunomodulatory effects of carvacrol against ovalbumin-induced asthma in rats. Life Sci..

[48] Bombeiro A.L., Pereira B.T.N., Bonfanti A.P., Oliveira A.L.R. (2020). Immunomodulation by dimethyl fumarate treatment improves mouse sciatic nerve regeneration. Brain Res. Bull..

[49] Hegazy-Hassan W., Zepeda-Escobar J.A., Ochoa-García L., Contreras-Ortiz J.M.E., Tenorio-Borroto E., Barbabosa-Pliego A., Aparicio-Burgos J.E., Oros-Pantoja R., Rivas-Santiago B., Díaz-Albiter H. (2019). TcVac1 vaccine delivery by intradermal electroporation enhances vaccine induced immune protection against *Trypanosoma cruzi* infection in mice. Vaccine.

[50] Pereira I.R., Vilar-Pereira G., Marques V., da Silva A.A., Caetano B., Moreira O.C., Machado A.V., Bruna-Romero O., Rodrigues M.M., Gazzinelli R.T. (2015). A human type 5 adenovirus-based *Trypanosoma cruzi* therapeutic vaccine re-programs immune response and reverses chronic cardiomyopathy. PloS Pathog..

[51] da Silva T.A., Oliveira-Brito P.K.M., de Thomaz S.M.O., Roque-Barreira M.C. (2020). Artin M: Purification and Evaluation of Biological Activities. Methods Mol. Biol..

[52] Garber J.C., Wayne Barbee R., Bielitzki J.T., Clayton L.A., Donovan J.C., Hendriksen C.F.M., Kohn D.F., Lipman N.S., Locke P.A., Melcher J. (2011). National Research Council (US) Committee for the Update of the Guide for the Care and Use of Laboratory Animals.

[53] (1999). Norma Oficial Mexicana NOM-062-ZOO-1999: Especificaciones para el Cuidado y uso de Animales de Laboratorio; Diario Oficial de la Federación: Mexico City, Mexico. https://www.gob.mx/cms/uploads/attachment/file/203498/NOM-062-ZOO-1999_220801.pdf.

[54] Urribarrí R.S. (1974). Estudio comparativo de dos Métodos para valoración Cuantitativa de la Parasitemia por Tripanosomas. KASMERA.

[55] Rodríguez-Morales O., Carrillo-Sánchez S.C., García-Mendoza H., Aranda-Fraustro A., Ballinas-Verdugo M.A., Alejandre-Aguilar R., Rosales-Encina J.L., Vallejo M., Arce-Fonseca M. (2013). Effect of the Plasmid-DNA Vaccination on Macroscopic and Microscopic Damage Caused by the Experimental Chronic *Trypanosoma cruzi* Infection in the Canine Model. Biomed. Res. Int..

[56] Barr S.C., Schmidt S.P., Brown C.C., Klei T.R. (1991). Pathologic features of dogs inoculated with North American *Trypanosoma cruzi* isolates. Am. J. Vet. Res..

